# Insight in HIV Integration Site Selection Provides a Block-and-Lock Strategy for a Functional Cure of HIV Infection

**DOI:** 10.3390/v11010012

**Published:** 2018-12-26

**Authors:** Zeger Debyser, Gerlinde Vansant, Anne Bruggemans, Julie Janssens, Frauke Christ

**Affiliations:** Molecular Virology and Gene Therapy, Department of Pharmacological and Pharmaceutical Sciences, KU Leuven, Herestraat 49–Bus 1023, 3000 Leuven, Flanders, Belgium; gerlinde.vansant@kuleuven.be (G.V.); anne.bruggemans@kuleuven.be (A.B.); Julie.janssens@kuleuven.be (J.J.); frauke.christ@kuleuven.be (F.C.)

**Keywords:** human immunodeficiency virus, integrase, integration site selection, LEDGF/p75, HRP-2, LEDGIN, block-and-lock, HIV cure

## Abstract

Despite significant improvements in therapy, the HIV/AIDS pandemic remains an important threat to public health. Current treatments fail to eradicate HIV as proviral DNA persists in long-living cellular reservoirs, leading to viral rebound whenever treatment is discontinued. Hence, a better understanding of viral reservoir establishment and maintenance is required to develop novel strategies to destroy latently infected cells, and/or to durably silence the latent provirus in infected cells. Whereas the mechanism of integration has been well studied from a catalytic point of view, it remains unknown how integration site selection and transcription are linked. In recent years, evidence has grown that lens epithelium-derived growth factor p75 (LEDGF/p75) is the main determinant of HIV integration site selection and that the integration site affects the transcriptional state of the provirus. LEDGINs have been developed as small molecule inhibitors of the interaction between LEDGF/p75 and integrase. Recently, it was shown that LEDGIN treatment in cell culture shifts the residual integrated provirus towards the inner nuclear compartment and out of transcription units in a dose dependent manner. This LEDGIN-mediated retargeting increased the proportion of provirus with a transcriptionally silent phenotype and the residual reservoir proved refractory to reactivation in vitro. LEDGINs provide us with a research tool to study the link between integration and transcription, a quintessential question in retrovirology. LEDGIN-mediated retargeting of the residual reservoirs provides a novel potential “block-and-lock” strategy as a functional cure of HIV infection.

## 1. Introduction

Despite significant improvements in therapy, the HIV/AIDS pandemic remains an important threat to public health. According to the World Health Organization (WHO), 36.9 million people are infected worldwide and 1.8 million new infections occurred in 2017 [1]. In 2017, 21.7 million people worldwide were receiving antiretroviral treatment. Expanded access to combination antiretroviral therapy (cART), in particular in economically disfavored regions, has reduced the overall incidence of HIV-related deaths to about 940,000 in 2017 compared to 1.9 million in 2004. However, the 90–90–90 HIV treatment targets of UNAIDS remain unmet, as in 2017, only 75% of people living with HIV in the world knew their HIV status; among those only 79% were receiving treatment and among the patients receiving treatment only 81% were virally suppressed [1]. Still, current treatments fail to eradicate HIV, as proviral DNA persists in long-living cellular reservoirs, leading to viral rebound whenever cART is discontinued. As a result, the current treatment for HIV is life-long and requires strict adherence to prevent selection of antiviral resistance, which inevitably occurs under suboptimal treatment conditions. In addition, increasing treatment coverage in resource-limited settings means facing enormous infrastructural, logistic, and financial challenges. Therefore, a continuous academic effort to increase the basic understanding of HIV molecular virology, and in particular its mechanisms to persist in infected cells, is of paramount importance to validate novel therapeutic strategies and targets to cure HIV infection [2].

## 2. The Hurdles on the Road Towards an HIV-1 Cure

**Figure 1:**
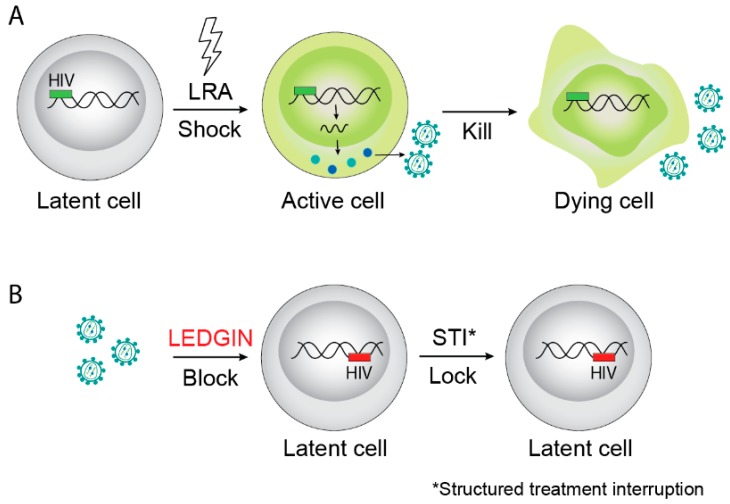
Examples of HIV cure strategies. (**A**) The “shock-and-kill” strategy aims to reactivate latent provirus followed by killing of activated cells by viral cytopathic effects on the host immune system. (**B**) The “block-and-lock” functional cure strategy aims to permanently silence latent reservoirs, for instance by LEDGIN-mediated retargeting of integration to sites that are less susceptible to reactivation after interruption of combination antiretroviral therapy (cART). LRA is latency reversing agents.

Even in patients on effective cART, a residual low-level viremia can be detected. In fact, the existence of a pool of persistent, replication competent HIV in patients on cART was demonstrated as early as 1997 [3,4]. However, studying HIV persistence has proven quite difficult. HIV DNA has been found in many anatomical sites, including blood circulating cells, the lymphoid system, the gut-associated lymphoid tissues, the brain, and other tissues [5,6], but it is not clear if replication competent virus can arise from all these sites. HIV can infect both myeloid and lymphoid cells and the relative contributions of different cell types to the reservoir remains a hot topic (reviewed in [6]). Additionally, two mechanisms can explain the persistent viremia in cART-treated subjects: ongoing low-grade viral replication owing to suboptimal tissue drug concentrations in so called sanctuary sites [7,8,9,10], and/or reactivation of HIV expression from pools of latently-infected cells [11,12,13]. In the literature, both sanctuary sites and latently infected cells are sometimes called reservoirs, since they both contain replication competent virus that can contribute to viral persistence and viral resurgence when treatment is interrupted. However, on the molecular level, latently infected cells contain intact, replication competent HIV DNA but do not produce new virus particles until reactivation occurs. Pre-integration latency refers to unintegrated HIV DNA that can still lead to expression of some viral proteins or integration upon stimulation, but its contribution to long-term persistence in patients treated with cART is considered to be limited [14,15]. In this review, we will focus on post-integration latency, where cells contain an integrated provirus that produces little to no viral mRNA, proteins, and/or progeny virus until reactivated. These latently infected cells are therefore not destroyed by viral cytopathic effects nor recognized by the immune system. However, upon reactivation of the provirus, transcription resumes, resulting in the completion of the viral replication cycle [15]. Since the focus of our review is on post-integration latency, we will thus define a reservoir as a certain pool of latently infected cells. To date, memory CD4+ T cells [16], including long-lived CD4+ T central memory stem cells (TCMs) [17] as well as T follicular helper cells (Tfh) [18], are considered to be the major subsets harboring the viral reservoirs. In addition to their long half-life, all memory CD4+ T cells are maintained through homeostatic proliferation [16] and they can also undergo clonal proliferation depending on their HIV-1 integration site [19,20,21]. Since expression of viral proteins is low or absent, these cells are not cleared by the immune system and can persist indefinitely. As such, memory CD4+ T cells provide a permanent source for virus reactivation and are likely responsible for the rapid rebound of plasma viral load observed after cART interruption. Therefore, the existence of latent reservoirs carrying replication competent provirus is the major obstacle towards finding a cure for HIV-1 infection [2]. This implies that a better understanding of viral reservoir establishment and maintenance is required to develop novel therapeutic strategies to target these latent reservoirs. Multiple strategies are being pursued [2,22]. So far, most efforts have been focused on the so-called ”shock-and-kill” strategy, where latency reversing agents (LRA), such as histone deacetylase (HDAC) inhibitors, are given to deliberately reactivate proviral transcription in latently infected cells [23] (Figure 1A). The hypothesis behind this approach is that reactivated cells will express viral proteins, allowing them to be recognized and destroyed by the host immune system. This strategy has entered exploratory clinical trials, but early results show limited evidence of efficacy [24,25,26,27,28,29]. Alternatively, autologous T- or stem cells can be modified to provide an HIV-1-resistant pool, e.g., by disrupting the *CCR5* gene with zinc-finger or CRISPR/Cas technology [30]. Furthermore, researchers are attempting to remove HIV-1 provirus from latent cells in cell culture using gene editing [31,32]. However, delivery of gene editing constructs to all reservoir cells in vivo remains a formidable hurdle. Moreover, all gene-editing strategies suffer from unknown risks due to off-target effects. A final strategy is to create a cellular reservoir resistant to reactivation, thereby preventing viral rebound [22]. This so-called “block-and-lock” strategy is the focus of this review (Figure 1B).

## 3. Molecular Determinants of HIV Latency

Multiple molecular factors determine HIV latency. Basic research on the cellular mechanisms that underlie the rate-limiting steps controlling viral gene expression in resting CD4+ T cells is required. Although latent HIV-1 proviruses are preferentially found integrated into actively transcribed genes [33,34], many HIV-1 proviruses display a transcriptional repression at the level of initiation and elongation, leading to establishment and maintenance of post-integration latency. The state of latency is associated with a block of transcription from the main HIV-1 promoter, the 5’ long terminal repeat (5’LTR). While HIV-1 Tat (trans-activator of transcription) activates viral transcription and limited Tat transactivation correlates with latency, other elements like epigenetic silencing and the absence of transcription factors are involved in the block of viral transcription (reviewed in References [12,22,35]). Moreover, not only HIV transcriptional silencing needs to be overcome to disrupt latent infection effectively. The link between the viral Rev protein, involved in HIV mRNA splicing and export, and latency is not well understood. Additionally, the role of cellular processes as translation, viral antigen expression, and/or processing in HIV latency, especially within resting CD4+ T cells, is not well defined. Furthermore, the potential role of exosomes in HIV-1 latency needs to be elucidated as they influence HIV infection and modulate immune responses by delivering viral proteins [36,37]. Recent evidence even suggests that exosomes from (un)infected cells can reactivate latent HIV [38,39,40]. Finally, a better understanding of host factors that enable or restrict latency is needed as well. In this review, we will focus on our understanding of the HIV-1 integration process and its possible link with transcription and latency.

## 4. HIV-1 Integration Is Mediated by LEDGF/p75, the “Global Positioning System (GPS)” of HIV

Whereas the integration step has been well studied from a catalytic point of view, it remains unknown how integration site selection and transcription are linked. In recent years, evidence has grown that the site of integration affects the transcriptional state of the provirus. Indeed, HIV integration is not random, but targeted towards the nuclear periphery and active transcription units in gene dense regions [34,41,42,43,44,45,46]. Interestingly, this pattern is highly conserved between all primate lentiviruses (simian immunodeficiency virus (SIV), HIV-1, and HIV-2) [47,48,49]. The observed differences between these viruses are small and involve the preferred orientation of integration (sense or antisense) and some integration hot spots. To achieve such specific integration patterns, lentiviruses use cellular cofactors. Lens epithelium-derived growth factor p75 (LEDGF/p75) in particular is a major determinant of HIV integration site selection (for a review see Reference [50]). In 2003, Cherepanov et al. identified LEDGF/p75 as a cellular binding partner of HIV integrase [51]. However, further research demonstrated that interaction with LEDGF/p75 is a conserved feature amongst all lentiviral integrases (including Feline Immunodeficiency Virus, Equine Infectious Anemia Virus, SIV, and HIV-2), pointing to its evolutionary importance. Additionally, this could explain the similarity between lentiviral integration patterns [52]. LEDGF/p75 is ubiquitously expressed and has been implicated in neuroepithelial stem cell differentiation and neurogenesis [53], lens epithelial cell gene regulation, stress responses [54], and regulation of gene expression during embryogenesis [55]. Next to its functions in cell biology, LEDGF/p75 has been associated with various pathological processes, such as autoimmune diseases [56], prostate cancer [57], mixed lineage leukemia [58], and HIV infection [51]. Both in health and in disease, LEDGF/p75 functions as a molecular tether, linking proteins and protein complexes with chromatin. As a chromatin reader, LEDGF/p75 recognizes nucleosomes associated with actively transcribed genes [59,60]. As such, it provides HIV with a molecular “global positioning system (GPS)”.

LEDGF/p75 is encoded by the PC4 and SFRS-interacting protein 1 (*PSIP1*) gene on chromosome 9 [54,61] and belongs to the hepatoma-derived growth factor related (HDGF) family [50]. Next to LEDGF/p75 and HDGF, this family contains four additional members (HDGF related proteins 1–4 (HRP1–4)). The HDGF family of proteins shares a conserved N-terminal HATH (homologous to the N-terminus of HDGF) domain with a characteristic structural domain: PWWP (Pro–Trp–Trp–Pro). The PWWP domain is known to bind methylated histones H3K36me2 and 3, DNA, and other negatively charged molecules, such as heparin. Next to the HATH domain, HDGF proteins contain a nuclear localization signal (NLS).

*PSIP1* encodes two isoforms, p52 and p75, resulting from alternative splicing [54,61]. Both splice-variants share their N-terminal region (amino acids 1–325), which harbors the PWWP domain, the nuclear localization signal (NLS), two AT-hook like motifs (ATH), and the supercoil recognition domain (SRD) [50]. LEDGF/p52 contains a C-terminal tail of only eight amino acids whilst LEDGF/p75 contains a largely unstructured region of an additional 205 amino acids. The only structured domain within this region is the integrase binding domain (IBD), named for its interaction with HIV integrase (IN) [62], although later work has demonstrated that many other cellular interaction partners also bind LEDGF/p75 through this domain [63]. The IBD domain of LEDGF/p75 interacts with the catalytic core domain (CCD) and the N-terminal domain (NTD) of integrase [62,64,65], and tethers the integration complex to the host chromatin [66]. This facilitates the integration into actively transcribed genes and ensures viral replication [67,68,69,70]. Next to its tethering function, LEDGF/p75 also stimulates the catalytic activity of IN and protects it from proteolytic degradation [51,71,72]. In human cells depleted of LEDGF/p75 by RNAi, in embryonic knockout mice-derived fibroblasts and in stable human LEDGF/p75 knock-down and knock-out cell lines, the absence of LEDGF/p75 significantly inhibits viral replication [67,68,69,70,73]. In the absence of LEDGF/p75, the related HRP-2 can substitute for its function [70,73]. The fact that overexpression of the IBD in cell culture effectively competes with endogenous LEDGF/p75 and potently inhibits HIV replication [68,74] provided proof-of-concept that the LEDGF/p75-integrase interaction indeed is a good target for antiviral therapy.

## 5. LEDGINs are Antivirals That Block the Interaction between HIV-1 Integrase and LEDGF/p75 and Display a Multimodal Mechanism of Action

In 2010, structure-based drug design targeting the interface between HIV IN and its cellular cofactor LEDGF/p75 led to the development of 2-(quinolin-3-yl)acetic acid derivatives that inhibit HIV-1 replication [75]. Since other inhibitors have been identified that bind to the same LEDGF/p75 binding pocket of HIV-1 IN, this class of antivirals is collectively referred to as LEDGINs [76]. The term LEDGINs is preferred over the term ALLINIs (allosteric integrase inhibitors), since not all allosteric integrase inhibitors bind to the LEDGF/p75 binding pocket [76]. LEDGINs abrogate the binding of LEDGF/p75 to HIV IN by binding to the IN dimer interface and allosterically inhibit the catalytic activity of IN (the so-called "early effect") [77,78]. Later, it was found that LEDGINs also inhibit late stage replication (the so-called "late effect") [77,79,80,81,82]. Viral particles produced in the presence of LEDGINs display morphological defects due to LEDGIN-induced IN multimerization. The majority of particles contain a delocalized ribonucleoprotein outside the capsid core or even lack a core. These crippled viruses are hampered during the next round of infection at the level of reverse transcription, nuclear import, and integration. Several pharmaceutical companies active in the HIV/AIDS field are exploring this novel class of inhibitors; for an in depth review we refer to Reference [76].

## 6. LEDGF/p75 Points the Way to a Block-and-Lock Strategy for a Functional Cure of HIV Infection

Since LEDGF/p75 is the main determinant of HIV integration site selection and since LEDGINs provide an elegant molecular tool to intervene with LEDGF/p75-mediated integration site selection, it was hypothesized that residual provirus after LEDGIN therapy may well be retargeted away from preferential integration sites. If so, this would open studies on the impact of retargeted integration on proviral gene expression and latency, and provide the rationale for a “block-and-lock” functional cure strategy (Figure 1B). In 2016, Vranckx et al. published a paper describing such experiments [83]. LEDGIN treatment clearly shifted residual HIV integration out of transcription units in a dose dependent manner [83]. An increased proportion of provirus integrated in the inverse orientation after LEDGIN treatment. In addition, the 3D localization of the integrated provirus shifted towards the inner nuclear compartment away from the nuclear periphery. Using the double reporter virus designed by the Verdin lab [84], it was shown that LEDGIN-mediated retargeting increased the proportion of provirus with a transcriptionally silent phenotype. Furthermore, this residual reservoir proved less susceptible to HIV reactivation. Of interest, almost simultaneously Chen et al. published a paper wherein a barcoded HIV vector (Barcoded HIV Ensembles (B-HIVE)) was used to experimentally prove that transcription is affected by the proximity of the integration site to genomic enhancers and that the extent of reactivation of the provirus with different LRAs is dependent on the integration site [85,86]. This finding corroborates previous findings whereby different HIV infected clonal cell lines express varying HIV RNA levels [41].

Many research questions remain to be answered. LEDGINs are known to interfere with HIV assembly. Does this so-called late effect also affect the transcriptional state after infection with the crippled virus? Of note, although LEDGIN treatment in cell culture partially shifts integration out of genes, no random integration is obtained. Still, transcription of residual provirus and reactivation of latent provirus can be completely suppressed by higher concentrations of inhibitor. This teaches us that fine tuning of the optimal chromatin environment of the provirus by LEDGF/p75 surpasses mere integration inside or outside of genes. Research is ongoing to elucidate the mechanism whereby LEDGF/p75 mediates optimal integration site selection. New methods, such as B-HIVE [85,86] and branched DNA imaging (bDNA) [87], can be used to study the link between LEDGIN-mediated retargeting and the transcriptional state of the provirus at the single-cell level. Furthermore, DNase or MNase assays can be used to investigate the role of nucleosome positioning [88]. Finally, the interaction between HIV capsid and cellular CPSF6 was recently proposed to bypass heterochromatin in the nuclear periphery to allow integration in the nuclear interior [89]. These data are at odds with reports on preferential integration in the nuclear periphery and thus require further investigation [44,45,46]. Another line of research focuses on the possible role of a complex containing LEDGF/p75, Iws1, and Spt6 in the regulation of HIV latency [90]. In conclusion, HIV may have evolved to use LEDGF/p75 as a molecular tether to ensure both a productive and a latent provirus population. In the absence of LEDGF/p75 the provirus may end up in a third deep latent population refractory to reactivation. 

## 7. Tat Inhibition Provides a Second Block-and-Lock Strategy

As discussed, interference with the GPS of HIV, LEDGF/p75, results in a deep latent state, an approach coined the “block-and-lock” strategy [22]. Next to targeting the integration event, a “block-and-lock” functional cure strategy that aims to inhibit HIV transcription is also being explored. Recent studies have shown that HIV transcription can be abrogated by inhibition of the trans-activator of transcription (Tat) [91,92,93]. Mousseau et al. first showed that the Tat inhibitor didehydro-cortistatin A (dCA), an analog of the natural product cortistatin A, selectively inhibits Tat transactivation of the HIV-1 promoter by binding to the trans-activating response (TAR)-binding domain of Tat [91]. Later, the same group demonstrated that dCA establishes a state of latency with a diminished capacity for reactivation. In a primary cell model of HIV-1 latency, dCA-induced inactivation of viral transcription persists even after drug removal, suggesting that the HIV-1 promoter is epigenetically repressed [92]. dCA mediates epigenetic silencing by increasing nucleosomal occupancy at Nucleosome-1, restricting RNAPolII recruitment to the HIV-1 promoter. The efficacy of dCA was also studied in the bone marrow–liver–thymus (BLT) mouse model of HIV persistence. Adding dCA to cART-suppressed mice systemically reduced viral mRNA in tissues. Moreover, dCA significantly delayed and reduced viral rebound levels upon treatment interruption, proving the validity of a ″block-and-lock″ strategy for obtaining a functional cure.

## 8. The Debate is Open

Can LEDGINs contribute to a functional cure? The intuitive response to this question is negative, because HIV reservoirs are established early after infection, preventing provirus retargeting strategies. Still, we now know that initiation of cART early after infection is effective in reducing the size of the viral reservoir and early treatment has now become standard care [94,95,96]. If early after infection a therapeutic window exists to reduce the size of the reservoir, addition of an antiviral to initial cART regimens that can modulate the functional reservoir would make perfect sense. Once LEDGINs are tested as antivirals in clinical trials for acute infection, follow-up of their reservoirs with proviral DNA loads and quantitative viral outgrowth assay (qVOA) analysis may be a wise thing to do. LEDGINs may also be beneficial when added to HIV pre-exposure prophylaxis (PrEP), as they might ensure that any residual infection under PrEP treatment results in a non-functional, deeply latent provirus.

With respect to chronic infection, in case no residual replication occurs after establishment of the viral reservoirs, LEDGINs can no longer modulate the functional reservoir. Yet, any residual replication at sanctuary sites with poor drug penetration by current drugs would be retargeted by LEDGIN treatment. Secondly, at present, the proportion of the functional reservoir that is mobilized upon treatment interruption is not known. Possibly, re-initiation of a cART regimen including LEDGINs after treatment interruption may modulate the functional residual reservoir if enough proviruses are mobilized and if not all replication is fully blocked. Although this is currently a speculative model, it should be testable once LEDGINs are evaluated in a clinical setting.

In conclusion, LEDGINs provide us with a formidable research tool to study the link between integration and transcription, a quintessential question in retrovirology. When LEDGINs enter clinical trials, adding diagnostic readouts to monitor their impact on the functional reservoir would address the question on a potential clinical use of the proposed “block-and-lock” strategy.
